# Rare Electrocardiographic Manifestation of Cystic Bronchiectasis in a 34-Year-Old Male

**DOI:** 10.14740/cr431w

**Published:** 2015-10-25

**Authors:** Santosh Kumar Sinha, Ramesh Thakur, Vikas Mishra, Amit Goel, Ashutosh Kumar, Mukesh Jitendra Jha, Chandra Mohan Varma, Pradyot Tiwari, Avinash Kumar Singh

**Affiliations:** a Department of Cardiology, LPS Institute of Cardiology, G. S. V. M. Medical College, Kanpur, Uttar Pradesh 208002, India

**Keywords:** Atrial overload, Bronchiectasis, Himalayan P-waves

## Abstract

Himalayan P-waves (amplitude > 5 mm) are often known to be classically associated with congenital heart diseases with right to left shunt like tricuspid atresia, Ebstein anomaly, combined tricuspid and pulmonic stenosis, ischemic heart disease, restrictive cardiomyopathy, etc., where they indicate a dilated right atrium and tend to be persistent. This type of P-waves is rarely seen in long-standing bronchiectasis and is usually transient. It can be easily confused with congenital heart disease. Here we report a case of Himalayan P-waves in patient with bronchiectatic lung disease which is a rare entity.

## Introduction

Bronchiectasis is a well known disease sequel to repeated pulmonary infections. This pathology affecting lungs can lead to functional and structural changes in the lung leading to various complications both functionally and structurally affecting basically through involvement of lung and heart, such as pulmonary hypertension and right ventricular and atrial overload. Himalayan P-waves have been reported in congenital heart diseases, but are rarely reported in chronic obstructive pulmonary disease (COPD) [1-3].

## Case Report

**Figure 1:**
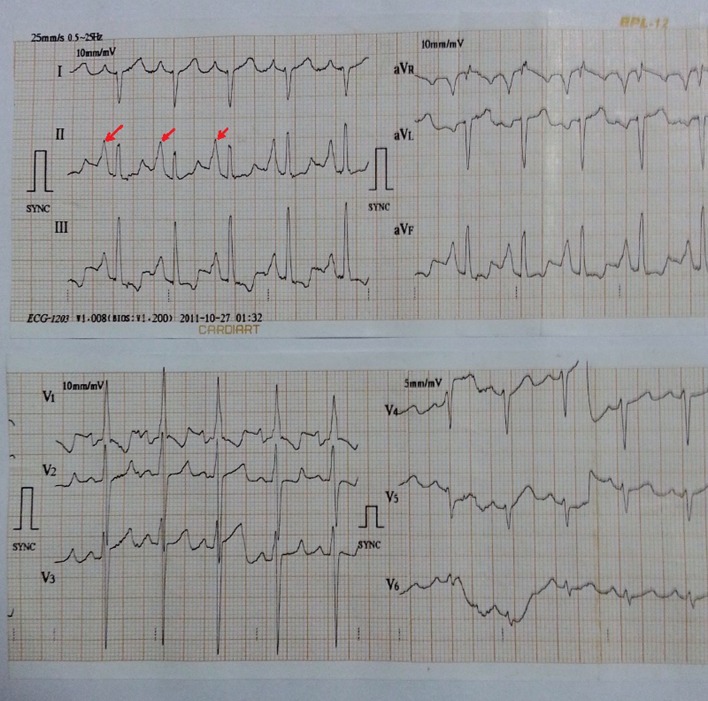
In lead II, III and aVF, P-wave was tall and peaked with maximum P-wave amplitude > 7 mm and larger than QRS complex in lead II. P-wave axis is +82°. Precordial leads also show right ventricular hypertrophy with strain.

**Figure 2:**
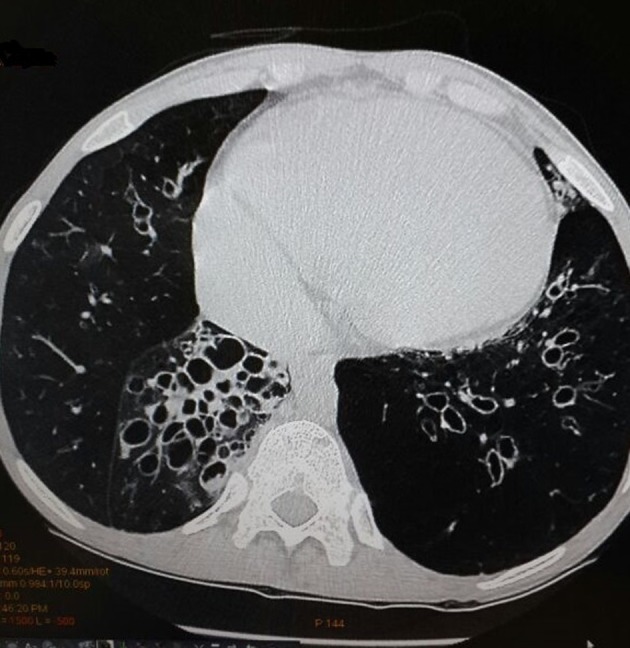
High-resolution computed tomography scan showing cystic bronchiectasis with bronchial wall thickening and mucous plugging.

**Figure 3:**
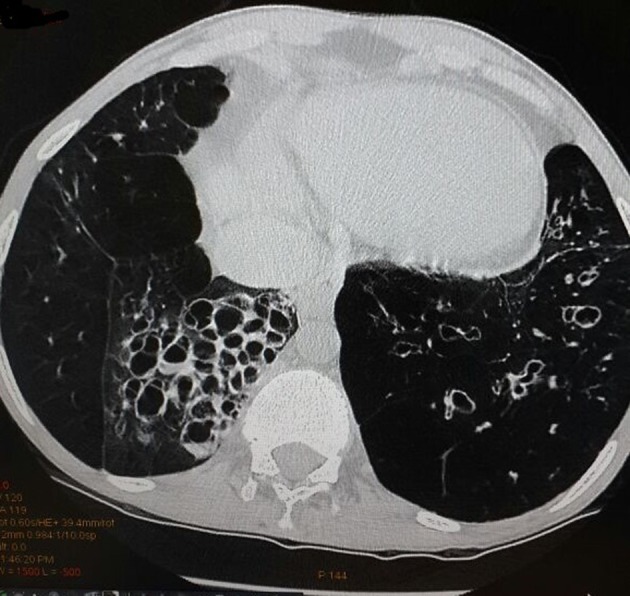
High-resolution computed tomography scan with cystic bronchiectasis showing bronchial dilation, lack of bronchial tapering and visibility of airways within 1 cm of the pleural surface (abutting the mediastinal pleural surface)

A 34-year-old male patient was admitted with complaints of cough and fever for 2 weeks. He had history of similar complaints in the past in the form of frequent episodes of cough with expectoration for which he was hospitalized and treated with intravenous (IV) antibiotics. He had no history of dyspnea, chest pain or pedal edema. General physical examination was unremarkable except for grade I clubbing. Clinical examination revealed blood pressure of 118/84 mm Hg in right upper limb in supine position with pulse rate of 96/min, regular, normal volume with a normal character with no radio-radial or radio-femoral delay and all peripheral pulses equally palpable. The jugular venous pressure was raised 2 cm above the angle of Louis with a prominent c-v pattern. The apical impulse was in the fifth intercostals space medial to the midclavicular line. The S1 was normal, and S2 was narrowly split with loud P2 component. There was pan-systolic murmur along the left lower sternal border radiating all over the precordium. On systemic examination, respiratory system showed barrel-shaped chest with anteroposterior and transverse diameter ratio of 1, widely spaced ribs and increased resonance to percussion, and breath sounds decreased in intensity symmetrically with prolonged expiration. Rest of the examination was normal. On investigations, total leukocytes were 14,200/mm^3^, polymorphs 72%, lymphocytes 22%, monocytes 4%, eosinophils 2%, hemoglobin 15.1 g%, platelets 211,000/mm^3^, ESR 14 mm first h and fasting blood sugar was 98 mg%. Blood culture was negative for any growth. Sputum culture showed streptococcus pneumonia sensitive to amoxicillin, and patient was treated accordingly. On chest radiography, there were bronchiectatic changes. Electrocardiogram showed normal sinus rhythm with heart rate of 102 and right axis deviation. In lead II, III and aVF, P-wave was tall and peaked with maximum P-wave amplitude > 7 mm and larger than QRS complex in lead II (Fig. 1). P-wave axis was +82°. This was referred as Himalayan P-waves suggestive of right atrial enlargement. Precordial leads showed slow R-wave progression with right ventricular strain pattern (Fig. 1). The 2D transthoracic echocardiography revealed enlarged right atrium and right ventricle with severe tricuspid regurgitation, pulmonary arterial hypertension and preserved ejection fraction. So these findings were consistent with sequelae of COPD. The findings of bronchiectasis were confirmed on HRCT chest as evident by cystic bronchiectasis with bronchial wall thickening and mucous plugging (Fig. 2). The bronchial dilation was also seen, lack of bronchial tapering and visibility of airways within 1 cm of the pleural surface, i.e., abutting the mediastinal pleural surface (Fig. 3).

## Discussion

Tall peaked P-wave as aptly characterized by Taussig as Himalayan P-waves (amplitude > 5 mm) has been classically reported in Ebstein anomaly [1]. It has also been described in congenital heart diseases with right to left shunt like tricuspid atresia [2], combined tricuspid and pulmonic stenosis [3] or acquired disease such as ischemic heart disease [4] or restrictive cardiomyopathy [5] where they indicate a dilated right atrium and tend to be persistent. Genesis of such P-wave has been ascribed to prolonged conduction in the enlarged right atrium [2]. Similar P-wave like ours has also been described in a case of emphysema by Chhabra et al [6]. The reason behind such Himalayan P-waves in these conditions is right atrial enlargement and strain secondary to pulmonary arterial hypertension subsequent upon destructive lung pathology. The patient’s low diaphragm position (related to long-standing secondary emphysema) and the severity of the right atrial hypoxia (directly related to severity of the bronchospasm) may also contribute [6]. Though patient had a long-standing history of repeated lower respiratory tract infections since childhood, but there was no evidence of congestive heart failure as evident by normal B-type natriuretic peptide on admission and normal left ventricular ejection fraction with moderate pulmonary arterial hypertension.
